# Genome-wide comparative analysis of NBS-encoding genes in four *Gossypium* species

**DOI:** 10.1186/s12864-017-3682-x

**Published:** 2017-04-12

**Authors:** Liuxin Xiang, Jinggao Liu, Chaofeng Wu, Yushan Deng, Chaowei Cai, Xiao Zhang, Yingfan Cai

**Affiliations:** 1 State Key Laboratory of Cotton Biology, College of Life Science, Henan Key Laboratory of Plant Stress Biology, Henan University, Kaifeng, Henan 475004 China; 2 College of Bioinformation, Chongqing University of Posts and Telecommunications, Chongqing, 400065 China; 3 United States Department of Agriculture, Southern Plains Agricultural Research Center, Agricultural Research Service, 2765 F & B Rd, College Station, TX 77845 USA

**Keywords:** *Gossypium* species, NBS-encoding gene, Amino acid sequence similarity, Gene structure, Disease resistance

## Abstract

**Background:**

Nucleotide binding site (NBS) genes encode a large family of disease resistance (R) proteins in plants. The availability of genomic data of the two diploid cotton species, *Gossypium arboreum* and *Gossypium raimondii*, and the two allotetraploid cotton species, *Gossypium hirsutum* (TM-1) and *Gossypium barbadense* allow for a more comprehensive and systematic comparative study of NBS-encoding genes to elucidate the mechanisms of cotton disease resistance.

**Results:**

Based on the genome assembly data, 246, 365, 588 and 682 NBS-encoding genes were identified in *G. arboreum*, *G. raimondii*, *G. hirsutum* and *G. barbadense*, respectively. The distribution of NBS-encoding genes among the chromosomes was nonrandom and uneven, and was tended to form clusters. Gene structure analysis showed that *G. arboreum* and *G. hirsutum* possessed a greater proportion of CN, CNL, and N genes and a lower proportion of NL, TN and TNL genes compared to that of *G. raimondii* and *G. barbadense*, while the percentages of RN and RNL genes remained relatively unchanged. The percentage changes among them were largest for TNL genes, about 7 times. Exon statistics showed that the average exon numbers per NBS gene in *G. raimondii* and *G. barbadense* were all greater than that in *G. arboretum* and *G. hirsutum*. Phylogenetic analysis revealed that the TIR-NBS genes of *G. barbadense* were closely related with that of *G. raimondii*. Sequence similarity analysis showed that diploid cotton *G. arboreum* possessed a larger proportion of NBS-encoding genes similar to that of allotetraploid cotton *G. hirsutum*, while diploid *G. raimondii* possessed a larger proportion of NBS-encoding genes similar to that of allotetraploid cotton *G. barbadense*. The synteny analysis showed that more NBS genes in *G. raimondii* and *G. arboreum* were syntenic with that in *G. barbadense* and *G. hirsutum*, respectively.

**Conclusions:**

The structural architectures, amino acid sequence similarities and synteny of NBS-encoding genes between *G. arboreum* and *G. hirsutum*, and between *G. raimondii* and *G. barbadense* were the highest among comparisons between the diploid and allotetraploid genomes, indicating that *G. hirsutum* inherited more NBS-encoding genes from *G. arboreum*, while *G. barbadense* inherited more NBS-encoding genes from *G. raimondii.* This asymmetric evolution of NBS-encoding genes may help to explain why *G. raimondii* and *G. barbadense* are more resistant to Verticillium wilt, whereas *G. arboreum* and *G. hirsutum* are more susceptible to Verticillium wilt. The disease resistances of the allotetraploid cotton were related to their NBS-encoding genes especially in regard from which diploid progenitor they were derived, and the TNL genes may have a significant role in disease resistance to Verticillium wilt in *G. raimondii* and *G. barbadense*.

**Electronic supplementary material:**

The online version of this article (doi:10.1186/s12864-017-3682-x) contains supplementary material, which is available to authorized users.

## Background

Cotton is one of the most economically important crop plants in the world and is the most important textile fiber crop worldwide. The most widely cultivated cotton species today are allotetraploid *Gossypium hirsutum* followed by *Gossypium barbadense*, both of which are originated from interspecific hybridization between the A-genome species *Gossypium arboreum* (A2) and the D-genome species *Gossypium raimondii* (D5) [1].

Verticillium wilt and Fusarium wilt are two of the most destructive diseases in cotton production worldwide. Verticillium wilt is caused by the soilborne fungal pathogen *Verticillium dahliae. G. raimondii* is nearly immune to the pathogen, and *G. barbadense* is usually resistant or highly resistant to *V. dahliae*, whereas *G. arboreum* and *G. hirsutum* are often susceptible to *V. dahliae* [2–8]. Fusarium wilt is caused by another soilborne fungal pathogen *Fusarium oxysporum* f. sp. *Vasinfectum. G. barbadense* is often more susceptible to *F. oxysporum* compared to *G. arboreum* and *G. hirsutum* [9, 10].

Resistance (R) genes play a central role in recognizing effectors from pathogens and in triggering downstream signaling during plant response to pathogen invasions [11, 12]. Numerous R genes from many plants have been cloned and characterized over the past few decades [13]. Most of the cloned R genes are nucleotide-binding sites (NBS) genes containing a NBS domain and constitute one of the largest plant resistance gene family [14, 15]. The NBS domain is part of the larger ~300 amino acid NB-ARC (Apaf-1, R proteins and CED-4) domain and contains five strictly ordered motifs including P-loop, kinase-2, kinase-3a, GLPL and MHDL [16, 17]. The NBS region binds and hydrolyzes ATP or GTP, and primarily works as a signal transduction switch following pathogen recognition [17]. NBS-encoding genes usually contain additional domains, TIR (the Toll/interleukin-1 receptor), CC (coiled-coil) or RPW8 (Resistant to powdery mildew in *A. thaliana*) in the N-terminal domain and LRR (leucine-rich repeat) domains in the C-terminal region [18].

NBS-encoding genes can be further classified into two major groups according to the presence or absence of different domains in the N-terminal region. The first group is comprised of proteins carrying TIR and members of the group are named TNL proteins (for TIR-NBS-LRR). The second, non-TIR-NBS-LRR group is usually known as CNL (for CC-NBS-LRR) and RNL (for RPW8-NBS-LRR), because most of its members encode CC or RPW8 in the N-terminal domain [19].

With the availability of genomic data for increasing number of cotton species, NBS-encoding genes could be systematically investigated to elucidate their role in contributing to the differences and relationships of disease resistances among cotton species, and help to decipher the mechanisms of disease resistance in cotton. The genome sequences of the two diploid cotton species, *G. raimondii* and *G. arboreum*, and the two allotetraploid cotton species, *G. hirsutum* (TM-1) and *G. barbadense*, have been reported [2, 20–22]. In the present work, the assembled genome sequences were utilized to identify NBS disease resistance genes for the four cotton species. Multiple approaches were utilized to assess these NBS genes’ architecture in the genome and their evolutionary history, including the characterization of functional domains, their distributions across the genome, their phylogenetic relationships and so on. The analysis provided genome level insights into disease resistance genes among cotton species, which can help to reveal the mechanism of disease-resistance and accelerate the disease-resistant breeding of cotton.

## Results

### Identification and classification of NBS-encoding genes

*G. arboreum*, *G. raimondii*, *G. hirsutum* and *G. barbadense* were selected to identify and compare NBS-encoding genes in their genomes. Searches with HMMER 3.1b2 in the *G. arboreum*, *G. raimondii, G. hirsutum* and *G. barbadense* genomes resulted in the identification of 246, 365, 588 and 682 NBS genes containing NB-ARC domain, respectively (Table 1 and Additional file 1: Table S1). The two allotetraploid cotton plants possessed almost twice the number of NBS genes compared to the two diploid cotton plants, probably because of hybridization between *G. arboreum* and *G. raimondii* without gene losses, or hybridization with rapid gene losses followed by gene replication after their divergence from the initial hybrid. It can also be due to the combination of the two processes.

**Table 1:** Classification and distribution of NBS-encoding genes in the four cotton genomes

Gene types	*G. arboreum*		*G. raimondii*		*G. hirsutum*		*G. barbadense*	
	Number	Percentage	Number	Percentage	Number	Percentage	Number	Percentage
CN	44	17.89%	39	10.68%	89	15.14%	92	13.49%
CNL	80	32.52%	107	29.32%	165	28.06%	143	20.97%
N	59	23.98%	62	16.99%	168	28.57%	171	25.07%
NL	53	21.54%	89	24.38%	154	26.19%	210	30.79%
RN	0	0.00%	1	0.27%	1	0.17%	2	0.29%
RNL	3	1.22%	3	0.82%	6	1.02%	9	1.32%
TN	2	0.81%	14	3.84%	0	0.00%	11	1.61%
TNL	5	2.03%	50	13.70%	5	0.85%	44	6.45%
Total	246	100.00%	365	100.00%	588	100.00%	682	100.00%

Additional domains, TIR, CC or RPW8 in the N-terminal region and LRR domains in the C-terminal region, were identified in the NBS-encoding genes. If a TIR, CC or RPW8 domain was detected before NB-ARC domain, it was coded as “T”, “C”, “R”, respectively; if LRR domain was detected after NB-ARC domain, it was coded as “L”. The NB-ARC domain was coded as “N”. The NBS-encoding genes were sub-classified into eight types according to their domain architecture (Table 1 and Additional file 1: Table S1): CN, CNL, N, NL, RN, RNL, TN and TNL. As shown in Table 1, *G. arboreum* possessed a larger proportion of CN, CNL, and N genes and a lower proportion of NL, TN and TNL genes than *G. raimondii*. For example, the proportions of CN, CNL and N genes of the diploid *G. arboreum* were 17.89, 32.52 and 23.98%, respectively, while the proportions of CN, CNL and N genes of the diploid *G. raimondii* were 10.68, 29.32 and 16.99%, respectively (Table 1). However, the percentage differences of RN or RNL genes between *G. arboreum* and *G. raimondii* were less than 1%, so the proportions of RN and RNL genes remained relatively unchanged. Similar results were observed between the two allotetraploid cotton species: *G. hirsutum* possessed a larger proportion of CN, CNL, and N genes and a lower proportion of NL, TN and TNL genes than *G. barbadense*, and the proportions of RN and RNL genes remained relatively unchanged. Therefore, with respect to the proportions of different NBS type genes, *G. arboreum* and *G. hirsutum* share similar distribution profiles, while *G. raimondii* and *G. barbadense* share similar distribution profiles. The results suggest that *G. hirsutum* may preferentially inherit NBS-encoding genes from *G. arboreum* progenitor, while *G. barbadense* may preferentially inherit NBS-encoding genes from *G. raimondii* progenitor. Moreover, the greatest percentage changes between *G. raimondii* and *G. arboreum*, and between *G. barbadense* and *G. hirsutum*, occurred in the TNL type genes, about 7 times. Therefore, these TNL genes may play a significant role in disease resistance in *G. raimondii* and *G. barbadense*.

In addition, the exon number of NBS genes was calculated based on the annotation information of cotton genomes (Additional file 1: Table S1 and Additional file 2: Table S9). NBS genes in *G. raimondii* and *G. barbadense* were predicted to have 2.8 and 3.8 exons in average respectively, which was much larger than the average number of NBS genes in *G. arboretum* and *G. hirsutum*, 2.3 and 2.2 respectively. The mean exon numbers in eight NBS gene type were quite different in each cotton species, but the exons of NBS genes with TIR or RPW8 domain were generally more than that of other NBS type genes, especially TNL type genes containing mean 4.4 to 9.5 exons. The larger exon number in cotton TNL type genes was consistent with the results of a previous study in the Arabidopsis, poplar, grapevine and *Rosaceae* [17, 23, 24].

### Chromosome location and gene cluster identification

**Fig. 1:**
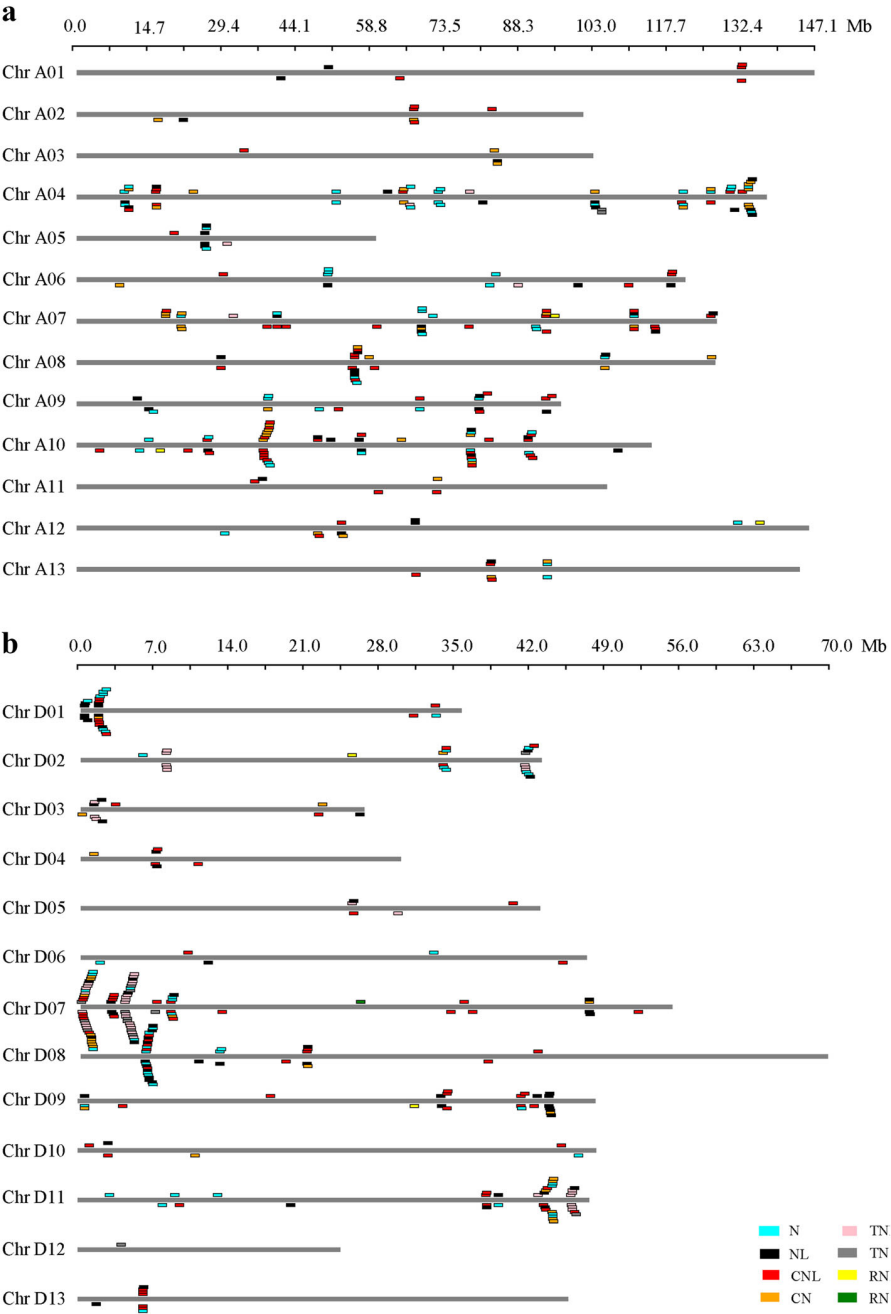
Distribution of NBS-encoding genes in chromosomes. **a** Distribution in *G. arboreum* chromosomes. **b** Distribution in *G. raimondii* chromosomes

Based on the location of individual NBS genes, 246 NBS genes and 273 of the 365 NBS genes, were mapped on the 13 chromosomes of *G. arboreum* (Chr A01-13, Fig. 1a) and *G. raimondii* (Chr D01-13, Fig. 1b), respectively; 311 of the 588 NBS genes and 395 of the 682 NBS genes were mapped on the 26 chromosomes (Chr A01-13 and Chr D01-13) of *G. hirsutum* and *G. barbadense*, respectively (Additional file 3: Figure S1A and B). The remaining NBS genes were located on other scaffolds that had not been yet linked to a chromosome (Additional file 1: Table S1).

The distribution of NBS-encoding genes was nonrandom among the chromosomes (Fig. 1, Additional file 3: Figure S1, Additional file 1: Table S1, and Additional file 4: Table S2). For example, *G.arboreum* chromosome A04 contained the greatest number (56) of NBS-encoding genes, including 6 of the 8 NBS gene types (N, NL, CN, CNL, TN and TNL); whereas only four NBS-encoding genes were located on chromosome A03 which contained 3 NBS gene types (NL, CN and CNL). *G. raimondii* chromosome D07 contained the greatest number (85) of NBS-encoding genes, including 7 of the 8 NBS gene types (N, NL, CN, CNL, TN, TNL and RN); whereas only one NBS-encoding gene was located on chromosome D12. *G. hirsutum* chromosome D09 contained the greatest number (86) of NBS-encoding genes, including 5 NBS gene types (N, NL, CN, CNL and RNL); whereas no NBS-encoding gene was located on chromosome D12. *G. barbadense* chromosome A11 contained the greatest number (70) of NBS-encoding genes, including 7 NBS gene types (N, NL, CN, CNL, TN, TNL and RNL); whereas no NBS-encoding gene was located on chromosome D04.

Moreover, the distribution of NBS-encoding genes was not even within the chromosomes and they tended to form clusters in other plants (tandem repeats [25–30]) (Table 2). This clustered arrangement has been thought to facilitate sequence exchange through recombinational mispairing [31]. Studies of NBS-encoding genes in *Arabidopsis*, rice, sorghum and maize also indicated uneven distributions on chromosomes and showed that most NBS-encoding genes were found in clusters [25–27, 32]. To identify NBS gene clusters, we used a previous definition [33] that a gene cluster was considered a chromosome or a scaffold region when such region contained two or more genes within 200 kb. Using this criteria, we identified 54, 52, 88 and 147 clusters containing 182, 287, 463 and 520 NBS genes in *G. arboreum*, *G. raimondii*, *G. hirsutum* and *G. barbadense*, respectively (Table 2, Additional file 1: Table S1, and Additional file 4: Table S2). The size of the clusters varied across the genome from 2 to 86 members (Additional file 4: Table S2). Each cluster had an average of about 3.4, 5.5, 5.3 and 3.5 genes and about 74.0, 78.6, 78.7 and 76.2% of NBS genes occurred in clusters in *G. arboreum*, *G. raimondii*, *G. hirsutum* and *G. barbadense*, respectively (Table 2). These results indicate that NBS-encoding genes of the four cotton species also tend to form clusters and the average NBS gene numbers per cluster among species were different (Table 2). Interestingly, though the total numbers of NBS-encoding genes among the four cotton species were quite different, their proportions of cluster genes remained relatively unchanged, 74-79% (Table 2). These proportions are greatly different from those of maize (57.0%) and sorghum (91.9%), but only slightly different from those of rice, *Arabidopsis or Medicago truncatula* (Table 2) [25]. We also identified 298 NBS-encoding genes and 52 NBS clusters in the genome of *Theobroma cacao*, a close relative of cotton (Additional file 1: Table S1). The 52 NBS clusters contained 241 NBS genes averaging 4.6 genes per cluster. About 80.1% of NBS genes occurred in clusters. Apparently, the proportion of NBS cluster genes in cacao was closer to that in cotton than to that in the other crops such as maize and sorghum. It was speculated that the duplication and clustering of NBS genes may be related to species evolution.

**Table 2:** Organization of NBS-encoding genes in ten plant genomes

Plant species	No. of NBS genes	No. of cluster genes	Proportion of cluster genes	No. of clusters	Mean members per cluster
*Gossypium arboreum*	246	182	74.0%	54	3.4
*Gossypium raimondii*	365	287	78.6%	52	5.5
*Gossypium hirsutum*	588	463	78.7%	88	5.3
*Gossypium barbadense*	682	520	76.2%	147	3.5
*Theobroma cacao*	298	241	80.1%	52	4.6
*Zea mays* (Maize)^a^	107	61	57.0%	22	2.8
*Sorghum bicolor* ^a^	236	217	91.9%	25	8.7
*Oryza sativa* (Rice)^a^	519	362	69.7%	104	3.5
*Arabidopsis thaliana* ^a^	171	125	73.1%	39	3.2
*Medicago truncatula* ^a^	469	310	66.1%	61	5.1

^a^Data from Cheng Y et al. [25]

### Similarity analysis of NBS-encoding genes among the four cotton species

Allotetraploid *G. hirsutum* and *G. barbadense* originated from interspecific hybridization between the A-genome species *G. arboreum* (A2) and the D-genome species *G. raimondii* (D5) [1]. To further elucidate the evolutionary relationship of NBS-encoding genes between the allotetraploid and the diploid cotton species, the NBS-encoding proteins with sequence similarity greater than 90, 80, 70, 60, 50, 40 and 30% between the allotetraploid and diploid cotton species were identified (Additional file 5: Table S3, Additional file 6: Table S4, Table Additional file 7: Table S5, Additional file 8: Table S6), respectively. The numbers of non-redundant NBS-encoding genes under different similarity level in *G. hirsutum*, *G. barbadense*, *G. arboreum* and *G. raimondii* were then calculated, respectively (Table 3). The results revealed that the number of non-redundant NBS-encoding genes in *G. hirsutum* or *G. barbadense* increased with decreasing sequence similarity, but the amount of increases became smaller (Table 3). For sequence similarity greater than 70%, the proportion of NBS-encoding genes in *G. hirsutum* or *G. barbadense* was greater than 75%. For sequence similarity greater than 50%, the proportion of NBS-encoding genes in *G. hirsutum* or *G. barbadense* was greater than 90%. Therefore, NBS-encoding genes of the allotetraploid cotton had high amino acid sequence similarity with that of the diploid cotton.

**Table 3:** Number of NBS-encoding genes and the corresponding proportions under different sequence similarity levels between the genomes for the four cotton species

Comparison between two cotton species (species A-species B)	Total no. of NBS genes in species A	Similarity >90%		Similarity >80%		Similarity >70%		Similarity >60%		Similarity >50%		Similarity >40%		Similarity >30%	
		No. of NBS genes in species A	Proportion of NBS genes in species A	No. of NBS genes in species A	Proportion of NBS genes in species A	No. of NBS genes in species A	Proportion of NBS genes in species A	No. of NBS genes in species A	Proportion of NBS genes in species A	No. of NBS genes in species A	Proportion of NBS genes in species A	No. of NBS genes in species A	Proportion of NBS genes in species A	No. of NBS genes in species A	Proportion of NBS genes in species A
*G.hirsutum* - *G.arboreum*	588	211	36%	392	67%	481	82%	509	87%	527	90%	537	91%	539	92%
▲*G.arboreum* - *G.hirsutum*	246	140	57%	202	82%	211	86%	214	87%	219	89%	221	90%	225	91%
*G.hirsutum* - *G.raimondii*	588	275	47%	452	77%	491	84%	522	89%	529	90%	537	91%	539	92%
▲*G.raimondii* - *G.hirsutum*	365	189	52%	259	71%	280	77%	284	78%	293	80%	296	81%	315	86%
*G.barbadense* - *G.arboreum*	682	194	28%	395	58%	521	76%	588	86%	616	90%	630	92%	642	94%
■*G.arboreum* - *G.barbadense*	246	115	47%	185	75%	212	86%	219	89%	222	90%	225	91%	226	92%
*G.barbadense* - *G.raimondii*	682	316	46%	509	75%	545	80%	615	90%	630	92%	643	94%	645	95%
■*G.raimondii* - *G.barbadense*	365	216	59%	310	85%	339	93%	345	95%	348	95%	350	96%	352	96%
*G.arboreum* - *G.raimondii*	246	87	35%	174	71%	207	84%	213	87%	215	87%	223	91%	225	91%
*G.raimondii* - *G.arboreum*	365	95	26%	237	65%	292	80%	325	89%	331	91%	345	95%	352	96%

The solid triangles (▲) represented the sequence similarities between *G. hirsutum* and the two diploid cotton species; The solid squares (■) represented the sequence similarities between *G. barbadense* and the two diploid cotton species

The numbers of NBS-encoding genes in *G. hirsutum* and *G. barbadense* were 588 and 682, respectively; about two times of the number of NBS-encoding genes in *G. arboreum* and *G. raimondii*, 246 and 365, respectively. However, the disease resistances of *G. hirsutum* and *G. barbadense* were not always stronger than *G. arboreum* and *G. raimondii*. The reason may be that most of NBS-encoding genes of *G. hirsutum* and *G. barbadense* had high amino acid sequence similarity with that of *G. arboreum* and G. raimondii, respectively (Table 3). Comparing sequence similarities between *G. hirsutum* and the two diploid cotton species, as signed with the solid triangles in Table 3, revealed that the NBS-encoding gene proportion of *G. arboreum* was greater than that of *G. raimondii* in *G. hirsutum* at all the sequence similarity levels. For example, 82% of NBS-encoding genes of *G. arboreum* compared to only 71% of the genes of *G. raimondii* had orthologous in *G. hirsutum* at greater than 80% sequence similarity level, indicating that *G. hirsutum* may inherit more NBS-encoding genes from *G. arboreum* than from *G. raimondii*. Similarly, comparing the sequence similarities between *G. barbadense* and the two diploid cotton species, as signed with the solid squares in Table 3, revealed that the NBS-encoding gene proportion of *G. raimondii* was greater than that of *G. arboreum* at all the sequence similarity levels. For example, 85% of NBS-encoding genes of *G. raimondii* compared to only 75% of the genes of *G. arboreum* had orthologous in *G. barbadense* at greater than 80% sequence similarity level, indicating that *G. barbadense* may inherit more NBS-encoding genes from *G. raimondii* than from *G. arboreum*. These evidences further support the findings of closely genetic relationships between *G. hirsutum* and *G. arboreum*, and between *G. barbadense* and *G. raimondii* from the study of the proportion comparisons of different NBS type genes in cotton species (Table 1).

The NBS-encoding proteins with sequence similarity greater than 90, 80, 70, 60, 50, 40 and 30% between *G. arboreum* and *G. raimondii* were also identified (Additional file 9: Table S7), respectively and the corresponding numbers of non-redundant NBS genes were calculated (Table 3). The proportion of similar NBS-encoding genes between *G. arboreum* and *G. raimondii* was also high (Table 3). For sequence similarity greater than 70%, the NBS-encoding gene proportions of *G. arboreum* and *G. raimondii* were more than 80%, indicating that these NBS-encoding genes were homogenous, and more tandem duplications of NBS-encoding genes may occur in *G. raimondii* than in *G. arboreum* after their divergence. There were 21 and 13 NBS genes (Additional file 10: Table S8) whose sequence similarity were less than 30% between the two genomes of *G. arboreum* and *G. raimondii*, respectively and they were all N type genes. These 21 and 13 NBS genes may play important roles in providing different disease resistance capabilities in *G. arboreum* and *G. raimondii*, respectively.

### Synteny analysis of NBS genes between diploid and allotetraploid cotton

**Fig. 2:**
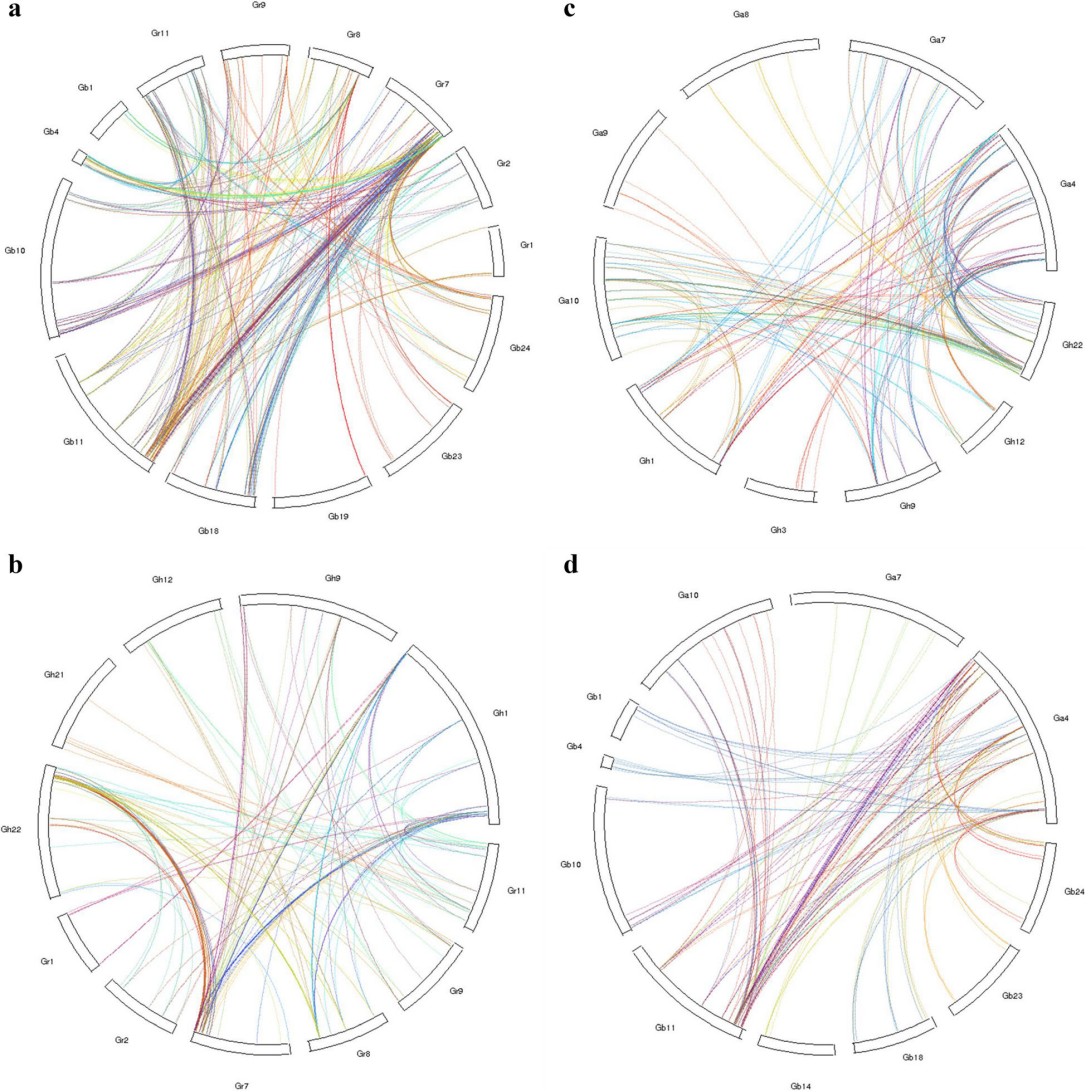
Synteny of NBS genes between the diploid and allotetraploid cotton. **a** Collinear gene pairs between *G. raimondii* and *G. barbadense*. **b** Collinear gene pairs of NBS genes between *G. raimondii* and *G. hirsutum*. **c** Collinear gene pairs of NBS genes between *G. arboreum* and *G. hirsutum*. **d** Collinear gene pairs of NBS genes between *G. arboreum* and *G. barbadense*

The synteny of genes across several plant species could provide insights to its evolution. The synteny analysis of NBS genes between diploid and allotetraploid cotton was conducted using McScanX (Fig. 2). There were 50, 30, 27, 20 syntenic blocks of NBS genes containing 378, 227, 189 and 154 collinear gene pairs between *G. raimondii* and *G. barbadense*, between *G. arboreum* and *G. hirsutum*, between *G. raimondii* and *G. hirsutum*, and between *G. arboreum* and *G. barbadense*, respectively (Additional file 11: Table S10). 157 (43%) and 88 (24.1%) NBS genes in *G. raimondii* were syntenic with that in *G. barbadense* and *G. hirsutum* respectively, while 115 (46.7%) and 62 (25.2%) NBS genes in *G. arboretum* were syntenic with that in *G. hirsutum* and *G. barbadense*, respectively. The results showed that more NBS genes in *G. raimondii* and *G. arboreum* were syntenic with that in *G. barbadense* and *G. hirsutum* respectively, indicating that the NBS genes between *G. raimondii* and *G. barbadense*, and between *G. arboreum* and *G. hirsutum* possessed closer evolution relationships, which was consistent with the results of similarity analysis and structural architectures analysis.

### Phylogenetic analysis of NBS-encoding genes containing TIR domain

**Fig. 3:**
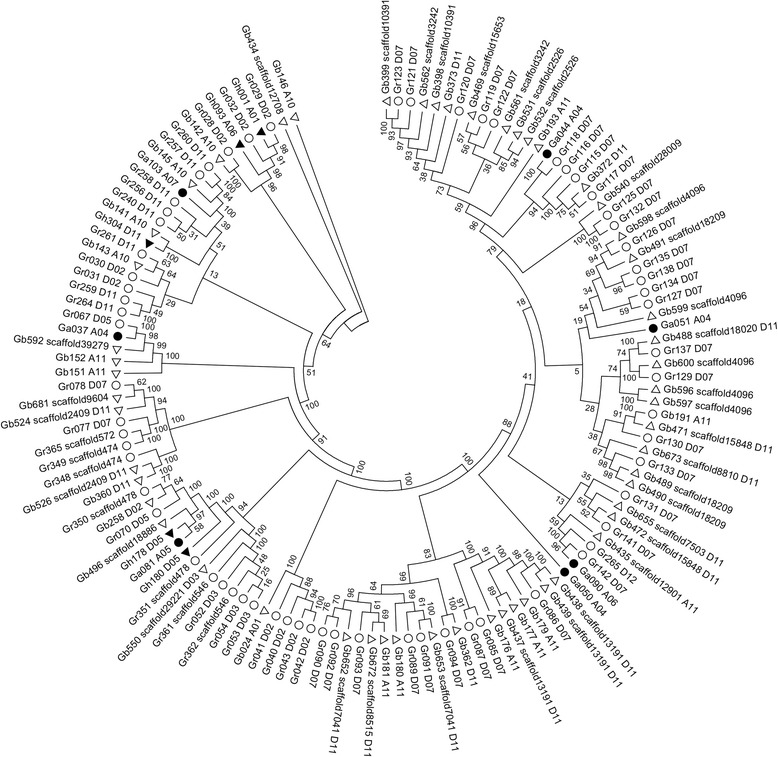
Phylogenetic tree derived from NBS-encoding genes containing TIR domain in *G. arboreum*, *G. raimondii*, *G. hirsutum* and *G. barbadense*. The neighbor-joining tree was constructed using the sequences of 131 TIR-NBS proteins in the four cotton species. Bootstrap values were indicated on the branches. Each NBS protein was labeled by its name (Additional file 1: Table S1). The *filled circles*, the *hollow circles*, the *solid triangles* and the *hollow triangles* represented the genes of *G. arboreum*, *G. raimondii*, *G. hirsutum* and *G. barbadense*, respectively

The amino acid sequences of the 131 NBS-encoding genes containing TIR domain (TIR-NBS gene) from *G. arboreum*, *G. raimondii*, *G. hirsutum* and *G. barbadense* were aligned, and a phylogenetic tree was generated by the Neighbor-joining method (Fig. 3).

Phylogenetic reconstruction of these TIR-NBS genes showed that the genes from the same cotton species were not always clustered together, but often scattered in different clades (Fig. 3). For example, the 7 TIR-NBS genes from *G. arboreum* were distributed in separate clades and were distantly related with each other. This result indicated that TIR-NBS genes among four cotton species were homologous. As shown in Fig. 3, Ga044_A04 gene in *G. arboreum*, and Gr115_D07, Gr116_D07, Gr117_D07 and Gr118_D07 genes in *G. raimondii* shared a common ancestor. Gr115_D07, Gr116_D07, Gr117_D07 and Gr118_D07 genes belonged to a gene cluster (Additional file 1: Table S1), indicating that tandem duplications of TIR-NBS genes occurred in *G. raimondii* after its divergence from *G. arboreum*. Except Gb434_scaffold12708 and Gb146_A10 which formed lone clades, the TIR-NBS genes of *G. barbadense* were closely related with that of *G. raimondii* (Fig. 3). For example, there were many sister genes between *G. raimondii* and *G. barbadense*, Gb399_scaffold10391 and Gr123_D07 genes, Gb469_scaffold15653 and Gr119_D07 genes, Gb142_A10 and Gr028_D02 genes and so on. This result indicated that *G. barbadense* inherited many TIR-NBS genes from *G. raimondii.*

## Discussion

The NBS-encoding gene family is the largest disease resistance gene family in plants and has been studied in many important plant species, including *Arabidopsis thaliana* [34], *Oryza sativa* [32], *Medicago truncatula* [35], *Zea mays* [25], and *Sorghum bicolor* [26]. Cotton is the most important textile fiber crop worldwide. Verticillium wilt and Fusarium wilt are the main threats to cotton production, and the immunity or resistance levels to the diseases differed among cotton species. Little is known about the mechanisms of resistance to Verticillium wilt and Fusarium wilt. To date, no comprehensive and systematic research has been conducted on the NBS-encoding genes in the four important species of the genus *Gossypium*. The present comparative study on genome-wide analysis of NBS-encoding genes in the four *Gossypium* species provided new insights and useful information.

Our bioinformatics analyses identified 246, 365, 588 and 682 NBS-encoding genes in *G. arboreum*, *G. raimondii*, *G. hirsutum* and *G. barbadense*, respectively. A different number, 355 NBS-encoding genes identified from *G. raimondii* by Wei et al. [36] may be due to the different criterions and a few genes containing NBS domain may be filtered out by programming firstly the homology alignment of protein sequences between all genes in G. raimondii and 113 reference disease resistance genes selected from plant resistance gene database [36]. The numbers of NBS-encoding genes of *G. hirsutum* and *G. barbadense* were about two times of that of *G. arboreum* and *G. raimondii*, but their disease resistance were not always stronger than that of *G. arboreum* and *G. raimondii*. It is suggested that the total number of NBS-encoding genes in the genome is not the decisive factor for the disease resistance in cotton. For example, *G. raimondii* is more resistant to Verticillium wilt than *G. hirsutum* [3–8], while *G. arboreum* is more resistant to Fusarium wilt than *G. barbadense* [9, 10]. Through the analysis of some resistance gene analogues encoding NBS-LRR domains in cotton, Khan et al. demonstrated that evolution and variation of NBS-LRR genes is one of the reasons for the susceptibility of *G. hirsutum* to the pathogens as compared to its donor parents [37]. The evolution and variation of NBS-encoding genes were selected for the survival of the allotetraploid cotton, so their resistances to different diseases were distinct. For example, *G. barbadense* usually is resistant or highly resistant to Verticillium wilt, whereas *G. hirsutum* is susceptible [3–8]; *G. barbadense* is more susceptible to Fusarium wilt than *G. hirsutum* [9, 10]. In our analysis, the disease resistances of the allotetraploid cotton were more closely related to the preferential adoption and duplications of their NBS-encoding genes from one of the two diploid parents. Thus, the comparative analysis of the proportions of different NBS type genes showed that *G. arboreum* and *G. hirsutum* had more NBS-encoding genes in common, while *G. raimondii* and *G. barbadense* had more NBS-encoding genes in common. For CN, CNL and N genes, the diploid *G. arboreum* had a greater proportion than the diploid *G. raimondii*, and the allotetraploid *G. hirsutum* had a greater proportion than the allotetraploid *G. barbadense*. Opposite results were observed for the NL, TN and TNL genes. The proportions of RN and RNL genes remained relatively unchanged. Exon statistics also showed that NBS genes in *G. raimondii* and *G. barbadense* generally possessed more exons than that in *G. arboretum* and *G. hirsutum*. Therefore, *G. hirsutum* may inherit more NBS-encoding genes from *G. arboreum*, while *G. barbadense* may inherit more NBS-encoding genes from *G. raimondii*. We found large proportion percentage changes of TNL and TN genes within the two diploid species and within the two allotetraploid species, especially TNL genes, indicating that TNL genes may have a significant role in disease resistance in *G. raimondii* and *G. hirsutum*. Li F et al. found similar differences in TNL and TN genes between *G. arboreum* and *G. raimondii*, and quantitative RT-PCR (qRT-PCR) analysis of the TNL and TN genes upon *V. dahliae* infection showed that there were different degrees of expression in the *G. raimondii* [2].

Our phylogenetic analysis of TNL and TN genes (TIR-NBS genes) revealed that the TIR-NBS genes of *G. barbadense* were closely related to that of *G. raimondii*, and *G. barbadense* inherited many TIR-NBS genes from *G. raimondii*. Further similarity analysis of NBS-encoding genes in the four cotton species showed that *G. arboreum* had a larger proportion of NBS-encoding genes similar to that of *G. hirsutum*, and *G. raimondii* had a larger proportion of NBS-encoding genes similar to that of *G. barbadense*, indicating that *G. hirsutum* may inherit more NBS-encoding genes from *G. arboreum*, while *G. barbadense* may inherit more NBS-encoding genes from *G. raimondii*. The synteny analysis showed that more NBS genes in *G. raimondii* and *G. arboreum* were syntenic with that in *G. barbadense* and *G. hirsutum* respectively, indicating that the NBS genes between *G. raimondii* and *G. barbadense*, and between *G. arboreum* and *G. hirsutum* possessed closer evolution relationships. These results are consistent with the research reported by He L et al. [38], Zhang T et al. [39], and Liu X et al. [40]. He L et al. demonstrated that the distribution of resistance gene analogue (RGA) of *G. hirsutum* between the two sub-genomes A and D of cotton was uneven, with RGA being more abundant in the A sub-genome than in the D sub-genome [38]. Zhang T et al. found that structural rearrangements, gene loss, disrupted genes and sequence divergence of *G. hirsutum* were more common in the A sub-genome than in the D sub-genome [39]. Liu X et al. revealed that A and D sub-genomes of *G. barbadense* had a high level of co-linearity with the *G. raimondii* genome [40]. The present comparative study of disease resistance in cotton also showed that *G. raimondii* and *G. barbadense* are immune or resistant to Verticillium wilt, whereas *G. arboreum* and *G. hirsutum* are often susceptible to the wilt [2–8]; *G. barbadense* is often more susceptible to Fusarium wilt than *G. arboreum* and *G. hirsutum* [9, 10]. Therefore, we propose that *G. hirsutum* inherited more NBS-encoding genes from *G. arboreum*, while *G. barbadense* inherited more NBS-encoding genes from *G. raimondii*, suggesting an asymmetric evolution of NBS-encoding genes. This will help to explain why *G. raimondii* and *G. barbadense* are similarly immune to Verticillium wilt, whereas *G. arboreum* and *G. hirsutum* are both susceptible to the wilt. Our study will help to reveal the mechanism of disease-resistance and promote the disease-resistant breeding to improve cotton disease resistance.

## Conclusions

In all, 246, 365, 588 and 682 NBS-encoding genes were identified in *G. arboreum*, *G. raimondii*, *G. hirsutum* and *G. barbadense*, respectively. The NBS-encoding genes tended to form clusters on chromosomes. There were many commonality of structural architecture, synteny and amino acid sequence similarity of NBS-encoding genes between *G. arboreum* and *G. hirsutum*, and between *G. raimondii* and *G. barbadense*, indicating that *G. hirsutum* inherited more NBS-encoding genes from *G. arboreum*, while *G. barbadense* inherited more NBS-encoding genes from *G. raimondii.* This suggests asymmetric evolution of NBS-encoding genes in the two allotetraploid cotton species. The number of NBS-encoding genes is not the decisive factor of disease resistance in cotton, and the disease resistances of the allotetraploid cotton is related to the preferential adoption and duplication of their NBS-encoding genes from one of the two diploid parents. This will help to explain why *G. raimondii* and *G. barbadense* are similarly immune or resistant to Verticillium wilt, whereas *G. arboreum* and *G. hirsutum* are similarly susceptible to the wilt, and the TNL genes may have a significant role in Verticillium wilt resistance in *G. raimondii* and *G. barbadense.*

## Methods

### Cotton genome resources

Four whole-genome sequenced cotton plants were used in the present study, including two diploid cottons (*Gossypium raimondii* and *Gossypium arboreum*) and two allotetraploid cottons (*Gossypium hirsutum* and *Gossypium barbadense*). *G. raimondii*, *G. arboreum* and *G. hirsutum* gene information was provided by the Cotton Research Institute, Chinese Academy of Agricultural Sciences (http://cgp.genomics.org.cn/), while *G. barbadense* gene information was provided by College of Plant Science and Technology & Group of Cotton Genetic Improvement, Huazhong Agricultural University (http://cotton.cropdb.org/cotton/). The information contains annotations of 40,976, 4,0134, 76,943, 109,918 protein-coding genes in the *G.raimondii*, *G. arboreum*, *G. hirsutum* and *G. barbadense* genomes, respectively. The genome sequences of *Theobroma cacao* were downloaded from CocoaGen DB (http://cocoagendb.cirad.fr./) which contained 46,143 protein sequences.

### Identification and classification of NBS-encoding genes

All predicted protein sequences from the cotton genomes and cacao genome were scanned with HMMER 3.1b2 [41] using the Hidden Markov Model (HMM) corresponding to the Pfam database (profile HMM library) (http://pfam.xfam.org/search#tabview=tab1; Expect value cut-off of default gathering threshold: the minimum score a sequence must attain in order to belong to the full alignment of a Pfam entry). All genes that contained NB-ARC domains (Pfam: PF00931) by the Pfam search were selected and considered as the NBS-encoding genes.

NBS-encoding genes usually have additional domains such as TIR, CC or RPW8 in the N-terminal domain and a variable number of LRR domains in the carboxy-terminal region [18]. The additional domains of these cotton NBS-encoding genes were also identified. The CC domain was detected using HMMER 3.1b2 [41] (https://www.ebi.ac.uk/Tools/hmmer/search/hmmscan) and the results were confirmed using program COILS (http://www.ch.embnet.org/software/COILS_form.html) with default parameters and 0.9 threshold [34]. The TIR, RPW8 and LRR domains were identified using the NCBI Conserved Domains Tool (https://www.ncbi.nlm.nih.gov/Structure/bwrpsb/bwrpsb.cgi; Expect value cut-off of default threshold: 0.01). All the sequences were searched against following databases contained in the NCBI Conserved Domains Tool: CDD v3.15, Pfam v28.0, SMART v6.0, KOG v1.0, COG v1.0, PRK v6.9 and TIGR v15.0. The following accession numbers were found for TIR: pfam01582, cl23801 and smart00255; for RPW8: pfam05659, and cl05301; and for LRR: pfam00560, pfam07723, pfam07725, pfam12799, pfam13306, pfam13516, pfam13855, pfam14850, cl19480, smart00370 and COG4886.

The annotation information of generic feature format (GFF) files for the genomes of four cotton species were used to calculate the exon number of NBS genes.

### Genome mapping and gene cluster analysis of NBS-encoding genes

Using positional information on chromosomes, cotton NBS-encoding genes’ physical positions were drawn with GenomePixelizer_October_01_2003 (http://niblrrs.ucdavis.edu/GenomePixelizer/GenomePixelizer_Welcome.html).

Previous studies showed that most NBS-encoding genes were arranged in clusters [25–27, 32]. Therefore, the clusters of NBS-encoding genes in cotton and cacao were identified based on the previously established criteria which considered a chromosome or a scaffold region as a gene cluster when two or more genes were located within a 200 kb region [33]. For example, for two adjacent genes in a chromosome or a scaffold, if the region between the first gene’s start location and the second gene’s the end location was less than 200 kb, then the two adjacent genes constitute a cluster.

### Alignment and similarity analysis of NBS-encoding proteins

Multiple alignments of *G. raimondii*, *G. arboreum*, *G. hirsutum* and *G. barbadense* NBS-encoding protein sequences were performed together using ClustalW [42] with default options and a nj format file containing genetic distance and matched length of any two sequences among all NBS-encoding proteins was generated. One minus the genetic distance was the similarity of two sequences. Considering amino acid sequence length of NB-ARC domain is about 300, two NBS-encoding gene sequences whose matched length was more than 200 amino acids were analyzed subsequently. First, the NBS-encoding genes whose protein sequence similarity is more than 90%, more than 80%, more than 70%, more than 60%, more than 50%, more than 40%, and more than 30% between *G. arboreum* and *G. raimondii* were identified using perl program, respectively. Then, the numbers of non-redundant NBS-encoding genes of *G. arboreum* and *G. raimondii* under different sequence similarity level were calculated, respectively. Similarly, the NBS-encoding genes under different sequence similarity level between *G. hirsutum* and *G. arboreum*, between *G. hirsutum* and *G. raimondii*, between *G. barbadense* and *G. arboreum*, and between *G. barbadense* and *G. raimondii* were also identified, respectively. The corresponding numbers of non-redundant NBS-encoding genes of cotton species under different sequence similarity level were then calculated.

### Synteny analysis of NBS genes between diploid and allotetraploid cotton

An BLASTP comparison with e-value 1e–05 using blast-2.2.24+ downloaded from NCBI provided the pairwise gene information (m8 format) between a diploid and a allotetraploid. According to the BLASTP output, the synteny analysis was constructed using McScanX (http://chibba.pgml.uga.edu/mcscan2/; default parameters) [43].

### Phylogenetic analysis of NBS-encoding genes containing TIR domain

Multiple alignments of *G. raimondii*, *G. arboreum*, *G. hirsutum* and *G. barbadense* NBS-encoding protein sequences containing TIR domain (TIR-NBS gene) were performed together using ClustalW [42]. A subsequent manual alignment correction was accomplished by using MEGA 5.05 [44]. Phylogenetic trees were constructed by means of the bootstrap neighbor-joining (NJ) method and the Kimura 2-parameter model that were provided by MEGA 5.05. The stability of internal nodes was assessed by bootstrap analysis with 1000 replicates. These trees were subsequently used to analyze the evolutionary relationships among TIR-NBS genes.

## Additional files

Additional file 1: Table S1. NBS-encoding genes information of *G. arboreum*, *G. raimondii*, *G. hirsutum*, *G. barbadense* and *T. cacao*. (XLS 495 kb)

Additional file 2: Table S9. Exon statistics in NBS genes and each NBS gene type in the four cotton species. (XLSX 9 kb)

Additional file 3: Figure S1. Chromosomal distribution of NBS genes in *G. hirsutum* and *G. barbadense*. A. Chromosomal distribution of NBS genes in *G. hirsutum*. B. Chromosomal distribution of NBS genes in *G. barbadense*. (DOC 3922 kb)

Additional file 4: Table S2. Information of NBS-encoding gene clusters of *G. arboreum*, *G. raimondii*, *G. hirsutum* and *G. barbadense*. (XLS 22 kb)

Additional file 5: Table S3. The amino acid sequence similarity between *G. arboretum* NBS genes and *G. hirsutum* NBS genes. (XLS 1623 kb)

Additional file 6: Table S4. The amino acid sequence similarity between *G. raimondii* NBS genes and *G. hirsutum* NBS genes. (XLS 2569 kb)

Additional file 7: Table S5. The amino acid sequence similarity between *G. arboretum* NBS genes and *G. barbadense* NBS genes. (XLS 1475 kb)

Additional file 8: Table S6. The amino acid sequence similarity between *G. raimondii* NBS genes and *G. barbadense* NBS genes. (XLS 3027 kb)

Additional file 9: Table S7. The amino acid sequence similarity between *G. arboretum* NBS genes and *G. raimondii* NBS genes. (XLS 409 kb)

Additional file 10: Table S8. The NBS-encoding genes whose amino acid sequence similarity less than 30% between *G. arboretum* and *G. raimondii*. (XLS 14 kb)

Additional file 11: Table S10. Synteny statistics of NBS genes between the diploid and allotetraploid cotton. (XLS 73 kb)

## Abbreviations

CC: Coiled-coil
Chr: Chromosome
CN: CC-NBS
CNL: CC-NBS-LRR
LRR: Leucine-rich repeat
NBS: Nucleotide binding site
NL: NBS-LRR
R: Resistance
RN: RPW8-NBS
RNL: RPW8-NBS-LRR
RPW8: Resistant to powdery mildew in *A. thaliana*
TIR: Toll/interleukin-1 receptor
TN: TIR-NBS
TNL: TIR-NBS-LRR
