# Supplementary material for: Genome-wide comparative analysis of NBS-encoding genes in four Gossypium species
Source: BMC Genomics. 2017 Apr 12;18:292. doi: 10.1186/s12864-017-3682-x (PMC5388996; doi:10.1186/s12864-017-3682-x)
Supplement: Supplementary file 1 [file 12864_2017_3682_MOESM3_ESM.doc]

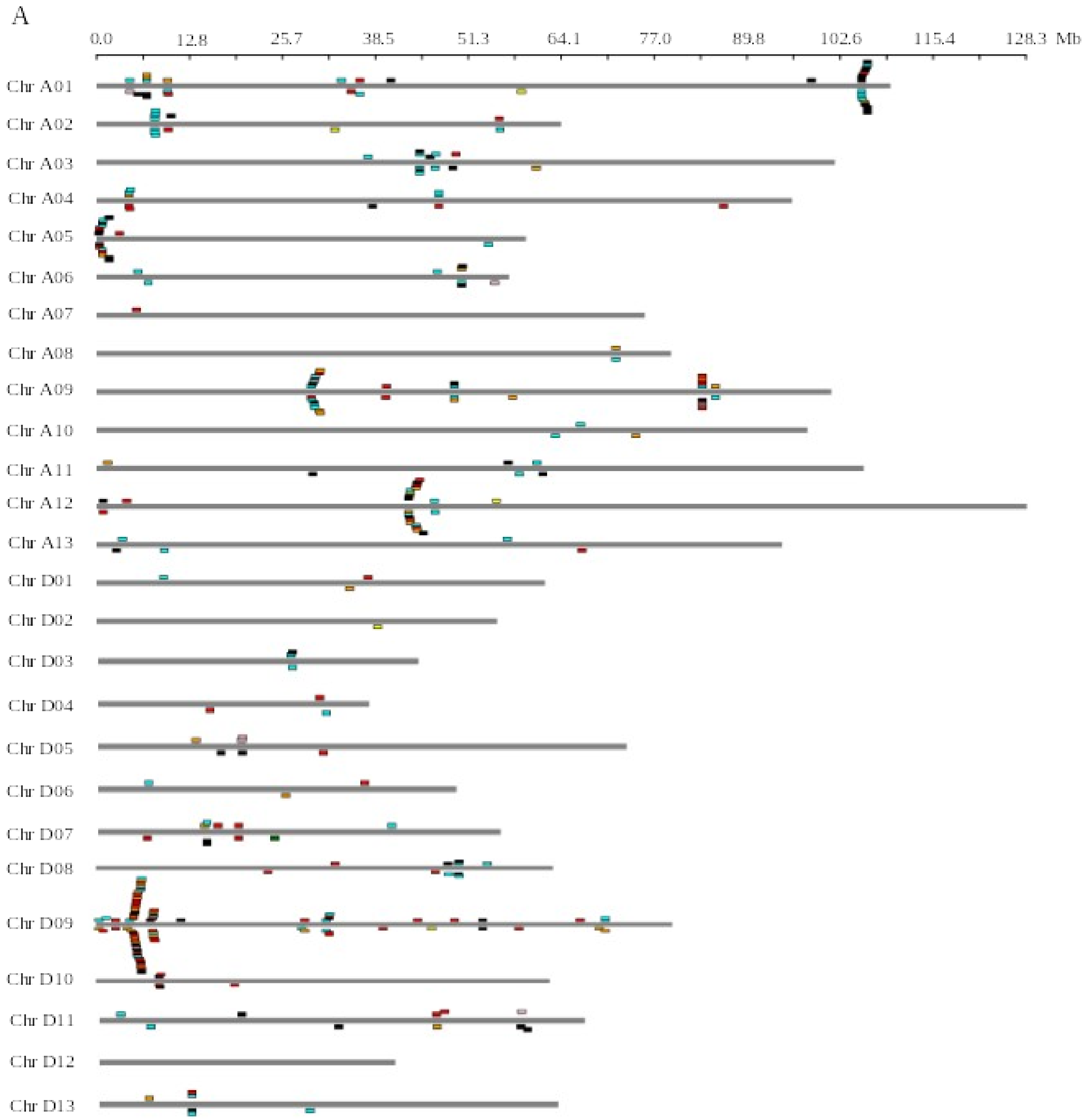

A
0.0
12.8
25.7
38.5
51.3
64.1
77.0
89.8
102.6
115.4
128.3 Mb
Chr A01
Chr A02
Chr A03
Chr A04
Chr A05
Chr A06
Chr A07
Chr A08
Chr A09
Chr A10
Chr A11
Chr A12
Chr A13
Chr D01
Chr D02
Chr D03
Chr D04
Chr D05
Chr D06
Chr D07
Chr D08
Chr D09
Chr D10
Chr D11
Chr D12
Chr D13

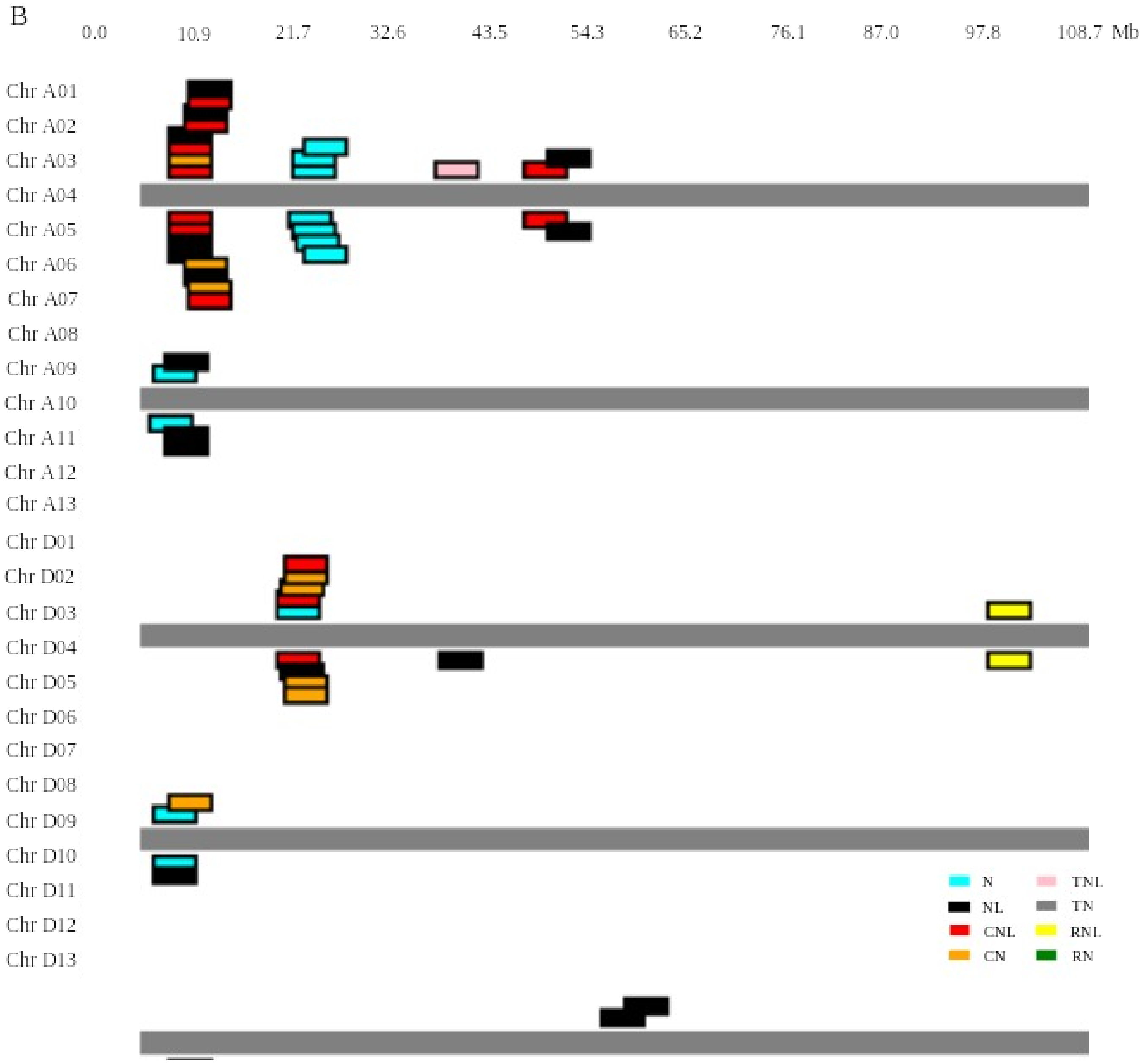
B
0.0
10.9
21.7
32.6
43.5
54.3
65.2
76.1
87.0
97.8
108.7 Mb
Chr A01
Chr A02
Chr A03
Chr A04
Chr A05
Chr A06
Chr A07
Chr A08
Chr A09
Chr A10
Chr A11
Chr A12
Chr A13
Chr D01
Chr D02
Chr D03
Chr D04
Chr D05
Chr D06
Chr D07
Chr D08
Chr D09
Chr D10
Chr D11
Chr D12
Chr D13
N
NL
CNL
CN
TNL
TN
RNL
RN
